# Exosomes as Emerging Biomarker Tools in Neurodegenerative and Neuropsychiatric Disorders—A Proteomics Perspective

**DOI:** 10.3390/brainsci11020258

**Published:** 2021-02-19

**Authors:** Boby Mathew, M. Shahid Mansuri, Kenneth R. Williams, Angus C. Nairn

**Affiliations:** 1 Yale/NIDA Neuroproteomics Center, New Haven, CT 06511, USA; boby.mathew@yale.edu (B.M.); kenneth.williams@yale.edu (K.R.W.); 2 Molecular Biophysics and Biochemistry, Yale University School of Medicine, New Haven, CT 06511, USA; 3 Department of Psychiatry, Yale University, 34 Park Street, 3rd Floor Research, New Haven, CT 06508, USA

**Keywords:** exosomes, biomarker, proteomics, neurodegenerative disease, neuropsychiatric disorders

## Abstract

Exosomes are synthesized and secreted by different cell types and contain proteins, lipids, metabolites and RNA species that reflect the physiological status of the cell of origin. As such, exosomes are increasingly being used as a novel reservoir for disease biomarker discovery. However, isolation of exosomes can be challenging due to their nonuniformity of shape and variable tissue of origin. Moreover, various analytical techniques used for protein detection and quantitation remain insensitive to the low amounts of protein isolated from exosomes. Despite these challenges, techniques to improve proteomic yield and increase protein dynamic range continue to improve at a rapid rate. In this review, we highlight the importance of exosome proteomics in neurodegenerative and neuropsychiatric disorders and the associated technical difficulties. Furthermore, current progress and technological advancements in exosome proteomics research are discussed with an emphasis on disease-associated protein biomarkers.

## 1. Exosome Biogenesis

Exosomes are a class of microvesicular bodies (MVB), 30–100 nm in size having a unique disc- or cup-shaped morphology [1]. After exosomes are synthesized by different cell types, they are secreted into, and transported through, various body fluids such as cerebrospinal fluid (CSF), blood, urine, and saliva. They are generated via an endosomal pathway in which the invagination of a cell membrane and encapsulation of cytosolic components results in the formation of early endosomes. These early endosomes undergo a maturation process through alterations in their biomolecular composition that leads to the formation of late endosomes. During this maturation process, the endosomal membrane invaginates to form intraluminal vesicles (ILVs) in the lumen of organelles. The microvesicular bodies then either undergo fusion with the lysosome membrane and follow a degradative pathway, or they fuse with the plasma membrane and are secreted into extracellular space [2]. The secreted ILVs are generally called exosomes or extracellular vesicles (EVs).

Exosomes are composed of various clinically important biomolecules such as proteins, lipids, nucleic acids, and metabolites that likely reflect the physiological status of the cell. Thus, the transfer of exosome content has the potential to affect intercellular communication under various physiological and pathological conditions [3,4]. Exosomes therefore could play an important role in intercellular interactions and in maintaining tissue homeostasis [5]. The endosomal sorting complex (ESCRT) and several other proteins are involved in the sorting and packaging of proteins during the maturation of exosomes. The molecular mechanisms that underlie the biogenesis of exosomes and protein complexes involved in cargo sorting and packaging have been described in several recent reviews [6,7,8] Here, we focus on the role of exosome proteins in neurodegenerative diseases and neuropsychiatric disorders. This review also describes the discovery and quantitative proteomic approaches used to identify central nervous system (CNS) and peripheral body fluid-derived exosomal protein biomarkers for various neuropathological diseases.

## 2. Role of Exosomal Proteins in Neurodegenerative Diseases

**Figure 1:**
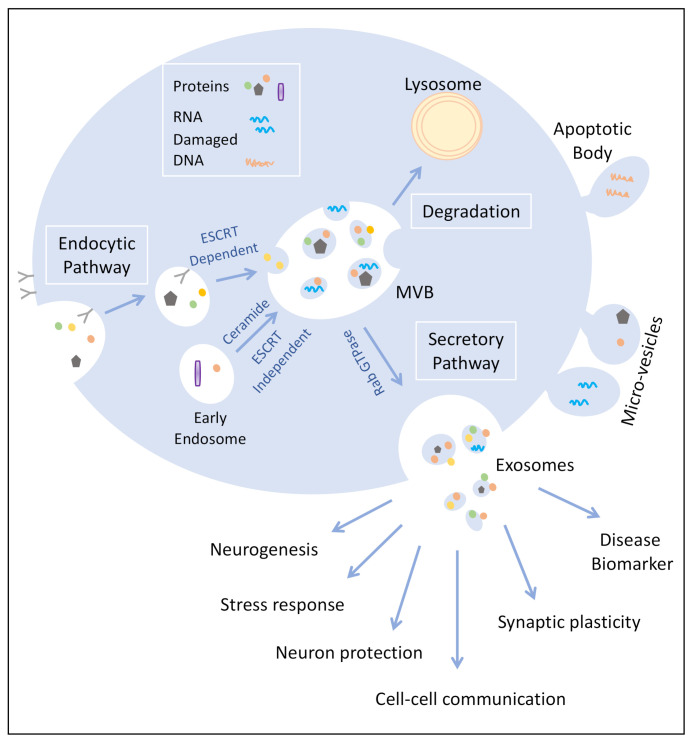
Depiction of the roles of exosomes in the CNS.

Exosomes are involved in cell-to-cell communication at multiple levels. Different subtypes of cells in the CNS such as astrocytes, microglia, oligodendrocytes, neurons, and Schwann cells produce cell type-specific exosomes that may act as cargo delivery vehicles mediating communication between different types of cells [9]. Exosome secretion regulated by neurotransmitters has been reported to assist in the communication between oligodendrocytes and neuronal cells and to play crucial roles in neuronal integrity and myelination [5]. Figure 1 shows a schematic representation that depicts potential functional roles of exosomes in CNS in the bidirectional flow of information between neurons and glial cells via the transfer of various biomolecules such as proteins, lipids, and nucleic acids. Exosomes may also play a key role in neuroprotection, including processes involved in cellular waste removal [10]. Alternatively, exosomes could mediate the neuron-to-glia or neuron-to-neuron transfer of toxic proteins [11,12]. For example, the majority of neurodegenerative disorders are driven by protein misfolding, seeded aggregation and cell–cell transmission of specific disease-related proteins that lead to the spread of pathological protein aggregates [11]. Some examples related to targeted analyses of proteins involved in neurodegeneration will be briefly discussed.

### 2.1. Alzheimer’s Disease

Although the detailed mechanisms remain to be elucidated, the trafficking and proteolytic processing of the amyloid precursor protein (APP) have been implicated in Alzheimer’s disease (AD) [13]. The role of exosomes in amyloid-β formation and its propagation into the extracellular milieu was first suggested by the studies of Rajendran et al. 2006, which showed that APP cleavage occurs in early endosomes and that amyloid-β peptides were present in exosome-associated microvesicles [14]. This latter observation was further confirmed by the association of amyloid-β aggregates with exosomes and by the finding that exosome bound Aβ-42 was colocalized with exosomal (CD63, CD9, and CD81) and neuronal (NCAM, L1CAM, and CHL-1) protein markers [15]. Multiple lines of evidence suggested that neuronally derived exosomes from AD patients have significantly higher levels of soluble Aβ-42 and its oligomeric aggregates compared to healthy controls [12]. The injection of astrocyte-derived exosome preparations into the so-called 5xFAD mouse brain (a mouse model of AD) increased aggregation of Aβ-42. Moreover, inhibition of neutral sphingomylenase-2 (nSMase2), a regulator of exosome biogenesis, by intra peritoneal injection of an nSMase2 inhibitor reduced Aβ-42 plaque levels in mouse brain [16]. These results are in agreement with other studies showing cargo proteins in exosomes purified from the brain were specifically enriched in APP-cleaving enzyme 1 (BACE-1), γ-secretase, soluble Aβ-42, soluble APP (sAPP)β, sAPPα, and APP C-terminal fragments [17,18]. These results strongly imply that exosome protein cargo may play a role in AD progression.

Exosomes have also been shown to have neuroprotective roles and to affect neurogenesis and cognitive impairment in amyloid-β animal models. One study used an AD mouse model generated by bilateral administration of Aβ-42 aggregates into the dentate gyrus [19]. Administration of mesenchymal stem cell (MSC)-derived exosomes into the same coordinates was shown to enhance neurogenesis and alleviate Aβ-42-induced cognitive impairment [19]. Another study showed that intravenous administration of MSC-derived exosomes tagged with CNS-specific rabies viral glycoprotein (RVG), which improved targeting to the cortex and hippocampus, decreased amyloid plaque deposition and amyloid-β levels [20]. Additionally, intravenous injection of RVG-tagged MSC-derived exosomes reduced the expression of pro-inflammatory factors (TNF-α, IL-β, and IL-6) and increased the expression of anti-inflammatory factors (IL-10, IL-4 and IL-13). Moreover, the RVG-tagged MSC exosomes reduced astrocyte activation and resulted in improvement of cognitive function in APP/PS1 mice [20].

The neuropathological accumulation of the microtubule-associated protein Tau, which leads to neurofibrillary tangles, is a widely explored phenomenon in AD pathology [21]. Secreted Tau with specific phospho-epitopes, AT270 (Thr(P)-181) along with exosomal protein markers, were observed in exosome fractions of M1C cells derived from neuroblastoma cells [22]. The CSF exosomes obtained from AD patients also showed similar phosphorylated forms of Tau suggesting these Tau forms are secreted through exosomes rather than being released from dead neurons [22]. A recent study revealed that EVs isolated from AD brain have significantly higher levels of Tau oligomers and protofibril forms of Tau as compared to prodromal AD (pAD) and control brains [23]. This study also suggested that EVs from an AD brain showed a higher uptake by cortical neurons and increased Tau seeding activity. The pathological effects of AD–EVs were predominantly observed in GABAergic interneurons, which resulted in a reduced intrinsic excitability of CA1 pyramidal cells and a reduction in synaptic markers [23]. Moreover, the perturbation of exosome biogenesis by the silencing or inhibition of sphingomylenase-2 resulted in a significant decrease in secretion and propagation of Tau in vitro and in vivo [24].

### 2.2. Parkinson’s Disease

Multiple studies have shown that α-synuclein and its aggregation in brain tissues play a significant role in the neuropathology of Parkinson’s disease (PD) [25]. Previous reports suggest that α-synuclein spreads from cell to cell leading to aggregation and neurodegeneration [26]. CSF exosomes isolated from PD patients have been found to be involved in the oligomerization of α-synuclein in a reporter cell line (H4 neuroglioma cells) where the seeding potential was significantly higher in PD–CSF exosomes compared to neurological controls [27]. Intracranial administration of brain-derived exosomes from patients diagnosed with dementia with Lewy bodies was found to induce α-synuclein aggregation in mouse hippocampal neurons and astrocytes [28]. An in vitro study demonstrated that exosomes isolated from the conditioned media of SH-SY5Y cells that overexpress α-synuclein could transfer α-synuclein to normal SH-SY5Y cells [29]. Administration of serum-derived exosomes from PD patients into mouse striatum led to accumulation of α-synuclein, ubiquitin, and P62, and also resulted in Lewy body-like protein aggregates [30]. This study also suggested that the observed elevated levels of inflammatory cytokines such as TNF-α and IL-1β in PD serum exosomes might trigger damage to dopaminergic neurons and PD-associated neurotoxicity [30]. In addition to neuron–neuron transmission, α-synuclein was also found to be propagated from neurons to astrocytes and microglial cells, and to induce a neuroinflammatory response in recipient cells [31,32]. Later studies by Chang et al. [33] found that microglial cells treated with α-synuclein enhanced exosome secretion and that these exosomes contained elevated levels of MHC class II molecules and TNF-α. When incubated with cortical neuronal cells these activated exosomes induced neuronal cell apoptosis that may be correlated with PD pathology. In a recent study, microglial cells were found to be preferentially targeted by exosomes isolated from PD patient plasma [34]. The PD-derived exosomes, containing a pathogenic oligomeric form of α-synuclein, resulted in proinflammatory responses and microglial activation [34]. Intriguingly, the intrastriatal inoculation of plasma-derived exosomes from PD patients in mice resulted in an initial uptake of exogenous α-synuclein by striatal neurons that over two weeks transferred to cortex and substantia nigra pars compacta (SNpc) [34]. Thus exosomes are involved in transport and propagation of pathogenic forms of α-synuclein from cell to cell, and likely this plays a crucial role in the neuropathology and disease progression of PD [35]. Although there are multiple lines of evidence suggesting plausible roles of exosomes in PD pathology, future studies aimed at how α-synuclein-containing exosomes are targeted between cells, and trigger mechanisms in α-synuclein seeding and aggregation and how this leads to PD need to be explored systematically.

### 2.3. Amyotrophic Lateral Sclerosis

Amyotrophic lateral sclerosis (ALS) is a motor neuron disease characterized by the propagation of motor neuron death from one focal motor unit to other adjacent cells possibly through altered neuron–glial cell communication [36]. Superoxide dismutase 1 (SOD1) was the first ALS associated enzyme. Gomes et al. 2007 [37] reported the presence of wild type SOD1 and its mutant form (G93A) in exosomes secreted by motor neuron-like cells (NSC 34 cells) that overexpressed SOD1. Later, exosome-mediated propagation of SOD1 was confirmed using SOD1 mutant-overexpressing cells [38]. Two types of propagation of human wild type SOD1 and mutant misfolded SOD1 have been proposed: (i) protein aggregates released from dying cells can be taken up via micropinocytosis, and (ii) secreted exosomes containing misfolded SOD1 [38]. Cytoplasmic aggregation of TDP-43, a DNA/RNA binding protein, is a hallmark of ALS pathophysiology [39]. In an in vitro study, it was shown that cell-to-cell transfer of TDP-43 can occur via exosomes or microvesicles. This study, which used neuronal cultures prepared in microfluidic chambers, also suggests both anterograde and retrograde trans-synaptic spreading of TDP-43 [40]. In a recent longitudinal study the levels of TDP-43 in plasma exosomes isolated from ALS patients were found to be significantly elevated at 3- and 6-month follow-ups [41]. These studies underline the potential role of exosomes in propagation of misfolded proteins and disease-specific protein aggregates in ALS disease onset and progression.

## 3. Isolation and Characterization of Exosomes for Protein Biomarker Discovery—Experimental Challenges

In the past decade, exosome research has witnessed several advances in the application of new methodologies for the isolation, purification, and molecular characterization of exosomes from biological fluids such as plasma, serum, urine, saliva, CSF, conditioned cell culture media, and tissues. The common methods of exosome isolation, along with their advantages and disadvantages, have been discussed in some recent reviews [42,43]. Table 1 summarizes various conventional and emerging methods for exosome isolation with their working principles, advantages, and disadvantages. Major challenges in exosome isolation are contamination with non-exosomal vesicles, co-isolation of protein aggregates and lipoproteins, damage to vesicle membranes, the requirement for specialized equipment and long preparation time. Different exosome isolation/purification methods have a varying impact on the yield, diversity, and downstream functions of recovered exosomes.

Characterization of purified exosomal fractions is generally based on various features such as size, shape, and protein or nucleic acid markers. Electron microscopy-based techniques such as transmission electron microscopy (TEM) and scanning electron microscopy (SEM) are the most commonly used techniques to characterize exosomes based on their size and morphology. These techniques provide higher accuracy and sensitivity (up to 0.1 nm to 3 nm) and are widely used for exosome characterization. The exosome concentration along with size and morphology can also be assessed by nanoparticle tracking (Nanosight™; NTA) [44]. This technique is based on the light-scattering characteristics of particles undergoing Brownian motion in solution. The hydrodynamic radius of each particle is calculated as a factor of its mean squared displacement and is displayed as a particle size distribution [45]. Though NTA is widely used in exosome characterization, the method suffers from a few drawbacks such as ambiguity in size determination due to the overlaying effect of larger vesicles masking smaller ones, and low fluorescent signal detection [46]. However, using electron microscopy based techniques in combination with NTA significantly improves the confidence of exosome characterization [47]. The total protein concentration also has been used as an index of exosome amount but the accuracy of this method depends on the variabilities induced by the isolation method and sample source, and the amount of contaminating non-exosomal proteins in the isolated fraction [48].

Recently, asymmetric-flow field-flow fractionation (AF4) was successfully used in fractionating subpopulations of exosomes from conditioned media obtained from various cell lines based on their density and hydrodynamic properties by two perpendicular flows (forward laminar channel flow and variable crossflow) [49]. This method separated two main exosome fractions, small exosome (ExoS) and large exosome (ExoL) vesicles with particle size 60–80 nm and 90–120 nm, respectively. Interestingly, this AF4 method reported another subpopulation termed as exomeres with particle size less than 50 nm and containing specific marker proteins [49]. Protein analysis of Exo-S and Exo-L indicate that Exo-S are most likely bona fide canonical exosomes that are enriched with proteins associated with endosomes, multivesicular bodies, vacuoles, and phagocytic vesicles. Exo-L may represent non-canonical exosomes or probably sEVs of different sub-cellular origin that are enriched with proteins associated with plasma membrane, cell–cell contact/junction, late-endosome, and trans Golgi network.

Immunoblotting is another commonly used molecular technique for exosome characterization that is based on the presence of specific exosomal marker proteins. The combination of cytosol and membrane-bound reporter proteins could improve the specificity of analysis. Proteins associated with the ESCRT complex (Alix, TSG101, HSC70, HSP90β and Flotillin-1), tetraspanin family (CD9, CD63, CD81), which are involved in exosome biogenesis and protein sorting, were found to be specifically enriched in vesicle preparations compared to the cell lysate [50,51]. A recent study compiled a detailed list of markers used for detection of exosomes from various biological preparations [52]. However, the lack of specificity of these markers leads to ambiguity in characterization; for example, tetraspanins have been identified in microvesicles and apoptotic bodies [53]. A recent study reported a new set of exosome specific markers syntenin-1, TSG101, ADAM10, and EHD4, that are consistently expressed in exosomes isolated from different cell types [54]. However, exosomal marker proteins can be unreliable in that all exosomes may not contain an equal number of marker molecules and the lack of a suitable loading control prevents accurate quantification. Since the purity of exosomal preparations also can be assessed by the lack of cell-derived organelle and apoptosis markers, such as GM130, PMP70, calreticulin and prohibitin; at least one protein from this category should also be quantified [55].

Fluorescence microscopy-based methods have been designed to provide non-invasive imaging of exosomes in vitro and ex vivo. Specifically, exosomes have been labelled using various methods: (i) staining using free dye targeting the lipid bilayer or genetically engineered fluorescent protein; (ii) using suitable affinity probes such as immunoglobulins or aptamers; (iii) co-expressing fluorescent proteins with exosome-specific proteins [56]. A recent study reported cell-based high-throughput exosome quantification using genetically labelled exosome marker proteins (CD63, CD9 and CD81) with high intensity luciferase NanoLuc (Nluc) [57]. Thus, by tracking fluorescently labelled exosome biomarkers it was possible to study the localization of exosomes, dynamics of biogenesis, exosome release and cellular uptake. However, this method often suffers drawbacks such as false-positive results caused by excess and free dyes and longer labelling and transfection time. In addition, bead-based methods have been developed for analyzing exosomes by flow cytometry [58]. Further, combining advanced imaging flow cytometry (iFCM) with a subset of specific markers enabled highly sensitive detection and multiparametric characterization of circulating exosomes in biological samples, and provided insight into their tissue origin [59]. Due to their small size, conventional flow cytometers lack detection capacity for exosomes [56]. However, high-resolution approaches are being developed and will be a focus of future exosomal analysis.

Irrespective of the methods used in their isolation, exosomes to be used in functional analysis or biomarker discovery should be of the highest purity possible without contaminating protein aggregates and molecules, especially when the differential expression of specific exosomal proteins is to be used as an indication of disease status. Because of the inherent drawbacks of purification techniques, it is very important to have a systematic comparison of the effect of different isolation techniques. Consistent application of isolation methods is also required to enable comparison of various studies of exosomal biomarkers.

## 4. Proteomics Approaches in Exosomal Protein Biomarker Discovery

With the caveats related to exosome isolation in mind, mass spectrometry-based proteomic methods are increasingly being used to complement and extend the more targeted approaches discussed above. Both exploratory and quantitative mass spectrometry analysis of exosomes isolated from different biological sources have revealed that they each contain specific sets of proteins rather than random cellular components. In addition to a conserved set of common proteins that are essential for vesicle biogenesis, structure, and trafficking, exosomes also contain proteins that are specific for the biological source and the particular experimental condition. Identification and characterization of these proteins provide crucial information regarding the molecular mechanisms that are involved in cargo sorting and trafficking, and clues about target cells [60]. Moreover, specific protein cargo identity and gene ontology will presumably allow recognition of the cell-type they originated from along with their physiological and pathological status [61]. An unbiased, non-hypothesis driven high-throughput proteomics analysis of exosomes will provide information about differential expression of proteins and their post-translational modifications. Such comparative knowledge of exosome proteomes from various clinical conditions could be valuable in developing potential biomarkers for early diagnostics, disease progression, prognosis, and treatment response [62]. Proteomic analysis of exosomes should therefore contribute significantly in developing minimally invasive diagnostics and next generation therapies. Some aspects of methodological considerations will be briefly reviewed, followed by discussion of application of proteomic approaches to the analysis of exosomes.

### 4.1. Mass Spectrometry-Based Exosome Proteomics

**Figure 2:**
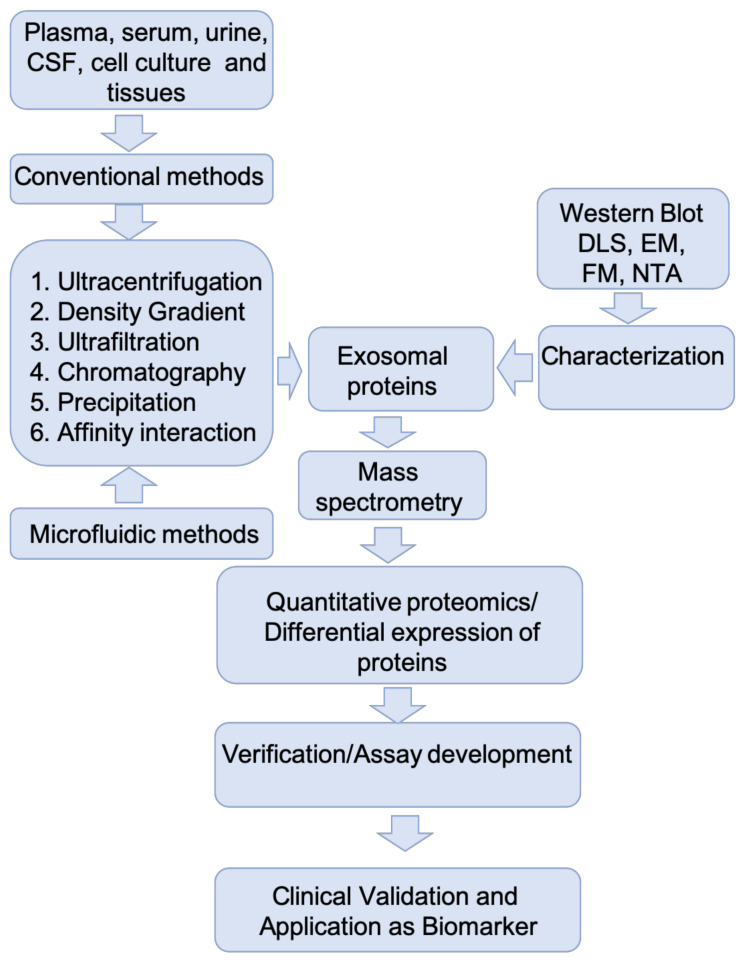
MS pipeline for identification and validation of protein biomarkers in exosomes for neurological disorders: nanoparticle tracking analysis (NTA), fluorescence microscopy (FM), electron microscopy (EM), dynamic light scattering (DLS).

Mass spectrometry (MS)-based proteomics (LC–MS/MS) has the potential to provide comprehensive analysis of exosome protein content. Figure 2 provides a schematic representation of a typical workflow used in MS-based proteomic analyses of exosomes. For the reasons discussed above, the purity of isolated exosomes is critically important in determining the outcome of proteomic analyses. Isolation methods should be optimized to obtain an effective separation of exosomes from other microvesicle subtypes and also to achieve minimal background from co-isolated protein aggregates and lipoproteins [63]. Since contaminants from preparation steps such as detergents, lipids, and polymeric materials can suppress the ionization and detection of low abundance peptides, specific clean-up steps such as protein precipitation and solid phase C-18 extraction should be used to reduce the concentrations of interfering contaminants [64]. Another issue is the presence of highly abundant proteins such as immunoglobulins and albumin in exosomal preparations from CSF and plasma that have significant ion suppressing effects on low abundance peptide ions [65]. Affinity-based depletion might be an effective strategy to remove non-specific abundant proteins from exosomal preparations. Alternatively, micro-size exclusion chromatography was found to improve identification and quantification of low abundance proteins from exosomes isolated from serum or plasma [66].

The standard bottom-up approach is the most commonly used method for MS-based exosome proteome analysis. Briefly, after exosome purification, proteins extracted from an exosome lysate are proteolytically digested and subjected to peptide fractionation through one dimensional liquid chromatography (1D LC) or two-dimensional liquid chromatography (2D LC). An efficient peptide fractionation reduces the sample complexity and helps in obtaining deeper protein coverage. Pre-analytical steps in bottom-up proteomics play a crucial role in proteome coverage and quantification. These pre-analytical methods are mainly two types; (i) gel-based and (ii) gel-free. The most widely used exosome sample preparation method for MS analysis is by separating the exosome proteome in 1D SDS–PAGE. Subsequently the gel bands are proteolytically digested and subjected to LC–MS/MS analysis [67,68,69,70]. For example, one 1D SDS–PAGE-based exosome proteome analysis using a medulloblastoma cell line identified 148 proteins [70] while a large-scale exosome proteomic analysis using human urine identified 1132 proteins [70]. In this latter study the 1D SDS-PAGE gel was divided from top to bottom into 40 slices that were each digested with trypsin and then subjected to LC–MS/MS analyses [68]. This 1D SDS-PAGE procedure has the advantage of removing contaminants that resulted from the steps involved in the exosome preparation. Another method of separating proteins prior to MS is by two-dimensional gel electrophoresis (2DGE). In 2DGE, the proteins are separated based on their isoelectric point in the first dimension and then they are subjected to a second dimensional separation based on molecular weight. This method has been used in multiple studies to reduce exosomal proteome complexity prior to MS analysis [71,72]. Although gel-based protocols provide excellent protein coverage, inherent limitations such as low reproducibility, time consuming steps, and low coverage for hydrophobic and membrane proteins limits their use in protein biomarker studies.

Recently, gel-free methods such as in-solution digestion and filter-aided digestion (FASP) protocols have gained interest in exosome proteome analysis. In the first method, following the lysis of membranes, proteins are reduced, alkylated and proteolytically digested using standard protocols [73,74]. Contaminants in the sample, such as detergents, urea, and salts are removed by C18 bead peptide enrichment and the eluted peptides are directly analyzed by LC–MS/MS. This method has major advantages in exosome analysis that include minimal sample loss and a lower sample amount requirement. A recent quantitative MS proteomic analysis of exosomes purified from donor hiPSC-derived neuronal cultures (that also used TMT isobaric tags for relative quantitation; see below) identified 2572 proteins [74]. The second method, FASP, is a modification of in-solution digestion in which proteins are trapped in a higher molecular weight cut-off filter and reduction, alkylation, and proteolytic digestion are achieved while keeping the proteins on top of the filter. This method allows the washing off of salt and detergent contaminants before digestion and an efficient elution of peptides after proteolytic digestion. Recent studies that used FASP methodology showed excellent exosome proteome coverage and proved this approach to be an effective digestion protocol [75,76]. One study identified and quantified 1630 proteins using FASP and an iTRAQ labeling technique (see below) from the serum of patients with pancreatic cancer [75].

### 4.2. Proteomic Quantification of Exosome Proteins

Protein identifications with reproducible and reliable quantitative data are critically important when assessing the extent of differential regulation of proteins and their potential as candidate biomarkers for a clinical condition. Quantitative high-performance mass spectrometric techniques extrapolate the protein identification obtained on the basis of unique peptide mass/charge ratio (*m*/*z*) to protein concentration based on peptide signal intensity. Quantitative proteomics can be broadly classified into two types based on the mode of acquisition and sample preparation: (i) label-free and (ii) labelled approaches.

For the label-free type, peptide ions are quantified based on their peak area (area under curve) or spectral count, which is observed in the mass spectra. Both these measures approximately correlate with the abundance of protein in a sample. Label-free quantification has been used in multiple studies to quantify exosome proteomes [77,78]. The major advantages of the label-free quantification approach are its flexibility in the number of samples that can be analyzed and the ease of sample preparation. In labelled approaches, proteins are modified with chemicals containing either stable isotopes or isobaric mass tags. The most common isobaric tag-based approaches are isobaric tags for relative and absolute quantification (iTRAQ), isotope-coded affinity tags (ICAT) and tandem mass tags (TMT) [79,80]. The range of unique isobaric mass tags in these techniques permits the multiplexing of samples, which significantly reduces operation-related sample-to-sample variations. Isobaric tags are reagents that have a reactive group that covalently modifies the peptides and a unique reporter group, whose abundance in the fragmentation spectra corresponds to the peptide concentration. Compared to label-free approaches, labelling strategies are often considered to be more accurate in quantitating protein abundances. However, the major drawbacks are limitation in the number of samples that can be analyzed in a single experiment and higher cost of the reagents [81]. Recent studies have used TMT [74,82] and iTRAQ [75,83] to identify potential exosome biomarkers for different clinical conditions.

Stable isotope labelling by amino acids in cell culture (SILAC) is a widely used strategy for metabolic labelling of proteins in cell culture. This technique is based on the principle of metabolically incorporating a stable isotope labelled ^13^C or ^15^N in lysine or arginine during protein synthesis. Quantification is based on the ratio of the intensity of each labelled peptide to its unlabeled counterpart (endogenous peptide). SILAC has been used in exosome protein biomarker studies [84,85]. SILAC is considered to be one of the best MS-based protein quantification strategies due to its high reproducibility and robustness. However, SILAC is designed to quantify proteins in cell culture samples and is not suitable for analysis of proteins in body fluids and tissue samples.

### 4.3. Proteomic Approaches for Identifying CNS Exosome Protein Biomarkers for Neurodegenerative and Neuropsychiatric Disorders

Exosomes are potentially a rich source of biomolecular cargo. Their ability to diffuse from the site of release and be retrieved from several biological fluids, where they may reflect pathological changes of cells present in relatively inaccessible tissues such as brain, makes them a promising target for biomarker discovery. As discussed above, multiple lines of evidence suggest that these vesicular bodies are implicated in age-associated neurodegenerative processes that may progress into cognitive impairment in later life. Thus, proteomic analysis of neural-derived exosomes (NDEs) isolated from CSF or serum/plasma could open a “window into the brain”, and might contribute to identification of biomarker candidates reflecting neuropathological mechanisms and disease progression [86,87,88]. Table 2 summarizes some of the exosome proteins used as biomarkers in studies of neurodegeneration. A recent quantitative proteomics study exploring the differential expression of proteins in exosomes derived from iPSC neurons transfected with mutant Tau (P301L and V337M) (mTau) showed significant alteration in protein expression compared to control exosomes [89]. Proteomic analyses identified 347 and 574 proteins in mTau and control exosomes, respectively. Eighteen proteins were unique to mTau exosomes, wheras 245 proteins were found only in control exosomes. Proteins unique to mTau exosomes included acidic nuclear phosphoprotein 32 family member A (ANP32A), AP-2 complex subunit α-1 (AP2A1), and V-type proton ATPase catalytic subunit A, that have been shown to be involved in synaptic dysfunction, memory loss and neuropathology [89]. Studies of exosome-mediated secretion of phosphorylated Tau from human neuroblastoma cells have shed light onto the possible role of exosomes in Tau pathophysiology in AD [90]. Quantitative proteomic analyses of exosomes from human neuroblastoma-derived M1C cells with wild type Tau expression found proteins specific to vesicle trafficking, signal transduction, tau oligomerization, and secretion. This study also reported elevated levels of phospho-tau (Thr(P)-181) in exosomes isolated from the CSF from AD patients later identified as having early stages of neurofibrillary changes reflecting AD pathology [22]. Another recent proteomic study found elevated levels of Thr(P)-181 tau in AD CSF and brain tissues [91]. Thr(P)-181 tau is an established marker for early onset of AD that is used as a CSF-based diagnostic for AD [91].

Another comprehensive study using label free quantitative proteomics coupled with a machine learning method reported a panel of four exosome proteins that could identify AD patients with 88% accuracy [92]. These biomarkers were annexin-A5 (ANXA5), NGF-induced growth factor (VGF), neuronal membrane glycoprotein M6-a (GPM6A), and alpha-centractin (ACTZ). A significant positive correlation was observed between GPM6A and pS396 Tau, and between GPM6A and Aβ-42 levels, while a significant negative correlation was found between VGF and Aβ-42 levels. Further validation using ELISA showed ANAX5 expression was significantly elevated in AD brain-derived exosomes as compared to controls. ANXA5 expression showed a positive trend with Braak stages of AD severity. Thus ANXA5 was suggested as a potential exosome biomarker that can differentiate AD from control exosomes and serve as a surrogate marker for Braak stages of AD progression [92].

Proteomic analysis of exosome-enriched fractions isolated from the CSF of sporadic ALS patients showed a significant differential regulation of various proteins [93]. Compared to controls, INHAT repressor (INR) protein was significantly up-regulated in CSF samples from ALS patients. However, this study did not provide sufficient evidence for the correlation of INR upregulation with ALS pathology [93]. Further studies exploring the role of INR in the pathophysiological mechanism of ALS is required. An intra-organ extracellular vesicle population isolated from the brain or spinal cord of a mouse model overexpressing human SOD1 with the G93A mutation (SOD1^G93A^) showed expression of SOD1^G93A^ in both the exosomal surface and lumen [94]. Quantitative proteomic analysis of brain-derived exosomes (BDEXs) from the SOD1^G93A^ mouse model and a non-transgenic mouse model showed that myelin–oligodendrocyte glycoprotein (MOG) was differentially regulated in both. The authors suggested that MOG might be a potential biomarker candidate to classify patients with early signs of ALS or neurologic dysfunction [94].

Another recent differential proteome analysis study of CSF exosomes from ALS patients showed that the proteasome-like Bleomycin hydroxylase, which has diverse protease activity, was significantly downregulated in ALS patients. Gene Ontology enrichment analysis demonstrated downregulation of the proteasomal core complex, endopeptidase complex, and carboxypeptidase activity that together support the hypothesis of alterations in protein homeostasis in ALS pathogenesis [121]. Further analysis of ALS patient subgroups showed that transmembrane glycoprotein (NMB), Protein–glutamine gamma-glutamyltransferase 2, Annexin 11, Ubiquitin-like modifier-activating enzyme 1, Cytochrome b-245 heavy chain and Cofilin-1 were differentially regulated in ALS patients with an expansion of the C9orf72 hexanucleotide repeat versus ALS patients who lacked this repeat [121]. Differential protein analysis of motor cortex extracellular vesicles (MCEVs) isolated from human postmortem ALS and neurological controls [122] found 16 proteins to be differentially regulated between these two groups that were involved in RNA binding, cell communication, transporter activity, signal transduction, and stress granule formation [122]. Both studies suggest the possible role of exosomes in the pathogenesis of ALS.

In a study linked to brain injury, proteomic profiling of EVs from the CSF of former National Football League (NFL) players identified the specific enrichment of proteins from neuronal cells, oligodendrocytes, microglia, and astrocytes [123]. Although the study failed to identify a potential biomarker for chronic traumatic encephalopathy compared to controls, the EV proteomes of the NFL players were enriched with proteins involved in AD pathology, age/telomere length ontology, and canonical liver/retinoid receptor activity [123]. Another study reported that extracellular microvesicles/exosomes isolated from the CSF of individuals with traumatic brain injury (TBI) had significant alterations in morphology, protein expression and the number of exosomes compared to controls [124]. An MS-based proteomic analysis from this study identified 91 proteins in MV/E from control CSF, whereas 466 proteins were identified in the counterpart from TBI CSF. Further annotation analysis identified cytoskeletal proteins (MAP2, HEATR5B, Syntaxin binding protein), and neurite outgrowth-related proteins (semaphorin-3C, Rho-related GTP-binding protein) to be unique to the TBI group. Systems biology and pathway analysis showed major pathways altered in TBI were complement activation, cell communication, synaptic endocytosis, cytoskeletal changes and microtubule cytoskeletal assembly. Notably, the levels of TBI protein biomarker candidates in human CSF MV/E such as intact αII-spectrin, SBDP150/145, SBDP120, intact GFAP, GFAP-BDP-38K, UCH-L1, and synaptophysin were able to successfully distinguish TBI from controls.

Proteomic analyses of exosomes are also being used in studies related to other CNS disorders. Rett syndrome, a neurodevelopmental disorder linked to autism, is caused by mutations in methyl-CpG-binding protein 2 (MECP2) [74]. Functional annotation analysis and differential proteomic analysis of purified exosomes from human-induced pluripotent stem cells (hiPSC) from a Rett syndrome patient with complete absence of MECP2 and isogenic control exosomes showed significant alterations in proteins related to neurogenesis and synaptic development [74]. Specifically, they contain signaling components for protein translation, axonal guidance, integrin signaling, ephrin signaling, and cytoskeletal regulation (Rho family, actin cytoskeleton), which have an impact on downstream signaling in neuritogenesis, development, morphogenesis and proliferation of neurons, and synaptic development and function [74].The isogenic controls, which were derived from the hiPSC neuronal culture from the same patient corrected for the MECP mutation, eliminated the proteome variability. Thus, this study provides evidence that the absence of even a single protein can result in a significant change in exosome protein cargo.

### 4.4. Proteomic Approaches to Identify Blood Exosome Protein Biomarkers for Neurodegenerative and Neuropsychiatric Disorders

Exosomes transport cargo molecules in a bi-directional manner—from the periphery to the brain as well as from the brain to the periphery—and across the blood–brain barrier (BBB). This makes them an attractive source of biomarkers originating in the CNS that can be isolated from peripheral body fluids [125]. The mechanism of exosome transport through the BBB is not clear; however, a few potential paths by which exosomes may cross the BBB are receptor mediated transcytosis, lipid raft-mediated micropinocytosis [126] and adsorptive mediated endocytosis [127]. But the underlying mechanism of exosome transport through the BBB is a subject of debate and needs further detailed study [128].

Compared to CSF biomarkers, blood biomarkers have the major advantage of sample availability and requiring less-invasive sample collection [129]. Recently, many studies have explored the potential of CNS-derived blood exosomes to identify protein biomarker candidates for neurodegenerative and neuropsychiatric disorders using immunoaffinity-based techniques [17,98]. For example, these studies have shown that neuron-derived exosomes (NDEs), or astrocyte-derived exosomes (ADEs), isolated from the blood of AD patients had significantly higher levels of previously reported AD pathology-related proteins (Phospho-T181-tau, Aβ-42, γ-secretase, soluble amyloid precursor protein (sAPP)β, and sAPPα). Notably, a recent study observed that, compared to free Aβ or total circulating Aβ in blood, exosome-bound Aβ reflected brain plaque distribution and provided better classification of AD patients [15].

A quantitative proteomics study of serum-derived exosomes from patients with PD at different stages identified various proteins to be differentially regulated compared to healthy controls [130]. Serum exosomes of patients with severe PD contained several unique proteins such as S100, tyrosine protein kinase receptor, lactoferrin, dermicidin, platelet activating factor acetyl hydrolase, and isocitrate dehydrogenase. Fourteen proteins that were differentially regulated between PD patients and controls were enriched in functional pathways such as prion diseases, ECM-receptor interaction, calcium signaling, and cAMP signaling, each of which has been implicated in PD pathology. However, a few limitations of this study, such as the relatively small sample size and the lack of validation of differentially expressed proteins, requires future work [130].

Potential biomarker candidates for chronic stress that can induce depression-like behaviors in rats were identified in serum-derived small extracellular vesicles (sEVs) [131]. Proteomic analyses identified differential expression of exosomal proteins in rat models exposed to repetitive stress by movement restriction either by restraint in small cages or immobilization in plastic bags, compared to control rats without stress [131]. The neurobiological difference between these stress models was established by reverting the depressive-like behaviors in rats induced by the stressors by antidepressant drugs (fluoxetine and reboxetine) acting on serotonin or noradrenaline mediated neurotransmission, respectively [131]. Functional interpretation of protein network analysis inferred that the common proteins found in the restraint and immobilization groups but absent in non-stressed animals were possibly related to stress- and depressive-like behaviors. The proteins identified in sEVs showed a significant overlap with exosome proteins isolated from primary astrocyte culture. Aldolase C, a differentially expressed protein when exclusively expressed with GFP tag in astrocyte progenitor cells was also identified in sEVs vesicles [131]. These findings support previous observations that the specific release of exosome cargo is a novel mechanism by which the brain communicates physiological and pathological status to the rest of the body [131].

## 5. Future Perspectives

Emerging studies suggest that exosomes play different roles in pathological CNS conditions by modulating transcription, neurogenesis, synaptic plasticity, neural circuit development, and neuroinflammation (see Figure 1). Their functions span actions such as serving as waste-disposal vehicles, as well as signal transmission vesicles, delivering neuroprotective agents to adjacent cells and responses to neurotransmitter cues. Exosomes have great potential as diagnostic tools for CNS disorders, especially because of their ability to traverse the BBB, thus providing the pathological and physiological status of the CNS. However, despite the large number of studies aimed at the identification of exosome biomarkers, only a few candidates have been identified that might qualify for diagnosis, progression prediction, and treatment of CNS disorders. Looking forward, large-scale systems biology approaches are required to explore the potential of exosomes as diagnostic biomarkers and to improve sensitivity and specificity for unambiguous classification of individuals with a given clinical condition from a healthy population. The use of sensitive and quantitative mass spectrometry-based proteomic approaches may be very helpful in this regard. Advancements in sample acquisition, ion detection, and protein quantitation in LC–MS/MS workflows will continue to improve the detection and analysis of low abundance proteins in exosomes. Coupled with improvements in consistent isolation and characterization methods, proteomic approaches have the capacity to distinguish specific proteins from contaminating proteins, aggregates and other vesicular bodies. The differentially expressed biomarker-candidate proteins and alterations in protein signaling pathways identified by high-throughput proteomic analysis can then be correlated to the pathophysiological mechanism of the clinical condition using functional assays in in vitro or in vivo models. Brain cell type-specific proteomic profiling of exosomal proteins and functional analyses will therefore play important roles in determining how specific biomolecular cargoes are packaged, targeted, and delivered, and it will allow early diagnosis, staging, prognosis, and disease intervention, thus alleviating the massive burden of these CNS disorders on the affected families and society. 

## Figures and Tables

**Table 1:** Methods used for purification of exosomes.

Methods	Advantages	Disadvantages
Ultracentrifugation	Simple, isolation from large samples	Equipment, contamination
Density gradient ultracentrifugation	High purity	Equipment, loss of samples
Ultrafiltration	High purity	Loss of sample, deformation of vesicles
Precipitation	Simple, preserves exosomes	Chemical contamination
Size-exclusion chromatography	Reproducibility, high purity, preserves exosomes	Co-isolation, equipment, sample volume
Microfluidic	High purity, fast	Device cost
Immuno capture	High purity, high selectivity	Antibody cost, nonspecific binding

**Table 2:** Exosomal protein biomarkers for CNS disorders.

Disease	Exosomes Source	Biomarker	References
Alzheimer’s disease	Plasma, CSF	Ab42, pT181-tau, pS396-tau, Total-tau, insulin receptor substrate 1, cathepsin D, LAMP, synaptophysin, synaptopodin, synaptotagmin-2 and neurogranin	[17,92,95,96,97,98,99,100,101,102,103]
Parkinson’s disease	Blood, CSF	alpha-synuclein, DJ1, clusterin, complement C1r subcomponent, and apolipoprotein A1, fibrinogen	[104,105,106]
Prion disease	CSF	PrP, Tau, 14-3-3, S100, Cystatin c, H-FAB	[107,108,109,110]
Fronto-temporal dementia	Blood, CSF	Nfl, Ab42, pT181-tau, pS396-tau, Total-tau	[98,111,112,113]
Amyotrophic lateral sclerosis	CSF, Plasma, Serum	TDP-43, Nfl, p-Nfh, SOD1, FUS	[114,115,116,117,118,119,120]
